# Parasite Extracellular Vesicles: Mediators of Intercellular Communication

**DOI:** 10.1371/journal.ppat.1004289

**Published:** 2014-08-28

**Authors:** Olivia Twu, Patricia J. Johnson

**Affiliations:** Molecular Biology Institute and Department of Microbiology, Immunology and Molecular Genetics, University of California, Los Angeles, Los Angeles, California, United States of America; University of Wisconsin Medical School, United States of America

## Introduction

The 2013 Nobel Prize in Physiology or Medicine recognized pioneering research on secretion of proteins outside the cell and the delivery of selected cargo to specific intracellular compartments via secretory vesicles. This seminal work, performed in yeast and mammalian cells, defined multiple vital roles vesicles play in cellular physiology. Recent studies have revealed the critical role of vesicles in intercellular communication. Here, we provide an overview of eukaryotic parasite-associated extracellular vesicles (EVs). Their contribution to host–pathogen interactions and possible use in vaccine design and disease diagnosis is discussed.

## What Are EVs?

EVs are vesicles released by cells that contain a complex mixture of proteins, lipids, glycans, and genetic information. Various names, including exosomes and microvesicles/microparticles, have been given to secreted EVs based on function, cell of origin, physical characteristics, contents, isolation method, and specific protein markers. Microvesicles, first discovered in 1967 [1], originate from the plasma membrane, have a diameter of 0.5–2 µm, and pellet at 10,000×g. Exosomes, first identified in the 1980s [2], [3], are derived from the multivesicular body, are 50–250 nm in diameter, and pellet at 100,000×g [4]. Both exosomes and microvesicles are released into the extracellular space with preserved right-side-out membrane topology. Current methods of vesicle isolation typically result in heterogeneous preparations that are enriched in one or more EV population(s). For a more thorough review of tools used to separate, evaluate, and analyze microvesicles and exosomes, see [5], [6]. For the rest of this review, exosomes and microvesicles will be collectively referred to as EVs.

## What Do EVs Do?

Interest in EVs began in earnest when their participation in various immune responses was demonstrated [7], [8]. Other critical functions, such as cell–cell signaling through fusion with target cells and delivery of components [9] soon followed, significantly increasing interest in the field. Skepticism that EVs were nonspecific, biologically insignificant “blebs” of random cargo released by stressed cells was diminished when it was shown that EVs affected specific pathways in target cells. EVs are now recognized as important vehicles that transport cargo from one cell to another, thereby mediating intercellular communication.

EVs may act to deliver molecular cargo between parasite and host cells. In this regard, they might be considered the functional equivalent of bacterial secretion systems [10], and are attractive candidates for allowing gene transfer between parasites and hosts. This has important implications for both pathogenesis and co-evolution. Once bound to a recipient cell, EVs can remain associated with the plasma membrane, dissociate, fuse, or be internalized [4]. Specific RNA cargo can be delivered via EV fusion, impacting RNA and protein expression and behavior of the recipient cell [9], [11]. In addition to RNA cargo, some EVs harbor DNA, retrotransposons, proteins, and camouflage viruses and viral components [4], [9], [12]. The content of EVs and their biological function depend on the origin and environment of the source and the target cell.

## Which Pathogens Secrete EVs?

While this review focuses on eukaryotic parasites, it is important to note that both fungi and bacteria secrete EVs that have been shown to carry potent virulence factors that aid in binding, invasion, cytotoxicity, release of toxins, and host immune modulation [13]. For more information on fungal EVs, see [14], and for a review on bacteria EVs, see [15]. EVs from eukaryotic parasites fall into two groups: those secreted from extracellular pathogens and those produced by human host cells infected by intracellular pathogens. Some pathogens can exist in both intracellular and extracellular forms and may produce vesicles in both states or result in increased host production of EVs in an intracellular state. Extracellular parasites such as *Trichomonas vaginalis*, *Trypanosoma cruzi*, *Leishmania* spp., and helminths [16]–[18] secrete EVs. Human cells infected with intracellular pathogens including the parasites *Plasmodium falciparum*, *Toxoplasma gondii*, and *Leishmania* spp. also secrete EVs containing both host and parasite proteins [19]–[24]. Ongoing research will most likely find that EV secretion is a common property of many pathogens.

## What Is Found in Extracellular Parasite EVs and What Do They Do?

Factors that regulate host gene expression or immunity are found in parasite-derived EV proteomes. These EVs contain pathogen-specific excretory and secretory proteins that often lack signal sequences, participate in delivery of virulence factors, and regulate virulence. For helminths [16] and *Leishmania* spp. [17], vesicular secretion may constitute up to 50% of a parasite's secretome. *T. vaginalis–*, *Leishmania* spp.– and *Try. cruzi*–derived EVs contain RNAs that may be delivered to host cells for regulation of host gene expression [17], [18], [25]. Both *T. vaginalis* and *Leishmania* spp. EVs are immunomodulatory and have other effects on host target cells to aid parasite infection [17], [18]. *T. vaginalis* EVs contain strain-specific contents, and EVs from more highly adherent strains can increase parasite attachment of less adherent strains to host cells through effects on both host and parasite [18]. *Leishmania* EVs can prime naïve immune cells in the local environment, for infection and contents of EVs vary depending on the parasite's environment [17]. Effects for parasite EVs have also been seen in vivo. Treatment of mice with *Leishmania* EVs prior to challenge with parasites exacerbates infection and increases immunosuppressive IL-10 production [26]. Similarly, injection of *Try. cruzi*–derived EVs prior to infection with the parasite increases tissue parasitism and inflammation in mice [27]. It has been hypothesized for several extracellular parasites that EVs act as messengers to prime host cells for parasite infection. Modulation of the host immune system in addition to parasite–parasite and parasite–microbiota interactions are active areas of EV research.

## What Do Intracellular Parasite-Induced EVs Contain and What Do They Do?

EVs derived from host cells infected with intracellular pathogens often contain a mix of parasite and host components. Moreover, similar to virus-infected cells, the EVs produced by parasite-infected cells contain altered host protein and RNA content relative to EVs secreted by healthy, non-infected host cells. EVs from virus-infected cells will not be discussed here; please see [28] for more information. Several human studies have shown that during infection with *Plasmodium* species, there are elevated levels of EVs produced by various host cell types [29]. EVs derived from erythrocytes infected with *P. falciparum* have been shown to aid in parasite survival, density sensing, transmission, and differentiation of gametocytes [22], [24], [29]. Furthermore, EVs can spread drug resistance, as those produced by transgenic *P. falciparum* parasites can transfer DNA encoding a drug resistance marker [24]. In addition to facilitating parasite communication, EVs induced by parasitized cells can impact host responses. Strong cytokine responses are elicited from host macrophages that internalize EVs produced by *P. falciparum–*infected blood cells [22]. EVs from *Toxoplasma gondii–*infected fibroblasts package a unique array of mRNA and miRNA transcripts that have the potential to modify neurologic activity [20]. EVs secreted by macrophages exposed to purified *Leishmania* exosomes can modulate expression of immune-related genes in naïve macrophages [30]. On the other hand, vesicles from parasite-infected host cells can broadcast infection status, resulting in enhanced host immune response [19]. Consequently, EVs from parasite-infected host cells might be useful as either biomarkers for diagnosis or as vaccines.

## Perspectives and Future Directions

EVs are effective mediators of intercellular communication, signaling, and gene regulation. Therefore, they are likely to be important players in determining the path of infection by pathogens. EVs are likely to aid pathogens in securing a niche in competitive environments. Acting as a pathogen's preliminary salvo to modulate host defenses, vesicles secreted by extracellular parasites can interact with host cells that may not otherwise be readily accessible to the parasite. It has been shown that EVs secreted by several parasites can be internalized by human host cells [16], [18], [22], [24], [26]. EVs may also be critical to maintaining parasite infection by aiding colonization and modulating the host immune response. The ability of pathogens to alter contents of EVs for various purposes is both underappreciated and understudied. Likewise, the potential of human host-derived EVs on infection has received little attention. Notably, it has been shown that *Try. cruzi* metacyclics can stimulate secretion of and fuse with human blood cell–derived vesicles to evade complement mediated lysis [21]. Whether host EVs affect parasite gene expression or behavior is an exciting area yet to be explored. Future research on the effect of EVs on host–parasite, parasite–parasite, and parasite–microbiome crosstalk is likely to uncover additional functions for secreted vesicles in these dynamic microbial communities.

The use of EVs as diagnostic, prognostic, and clinical tools offer promising new research applications. Vesicles are easily isolated from blood, urine, saliva, and other bodily fluids [4]. Thus as secreted EVs contain the protein or RNA signatures of pathological or physiological states of their source cell, analyses of these vesicles could enable non-invasive monitoring of pathogen response to newly developed drugs in animal models or typing of parasite infections. For example, alterations of the size and contents of bacterial EVs in antibiotic-treated animals can been detected by analyzing fluids from infected hosts [13]. Further investigation of EV contents and release in various environmental conditions will guide the potential use of EVs to characterize infectious states.

Another exciting potential use of EVs is for vaccines. EVs from parasites or those from host cells loaded with proteins and RNA derived from pathogens can be used as vaccine delivery systems. In the case of *Plasmodium yoelli* [23] and *Toxoplasma gondii* [31], parasite EVs or EVs from infected host cells can protect naïve animals from infection. Thus, EVs may serve as both biomarkers for infection as well as vaccine candidates. We have only scratched the surface of understanding the functions of EVs in infection biology. This glimpse forecasts that these specialized vesicles have several intriguing properties yet to be revealed.
